# Innate immune profiling reveals a specific reduction of CD57^+^CD62L^+^CD161^+^ NK cells in CMV-positive males with hypertension

**DOI:** 10.3389/fimmu.2026.1749702

**Published:** 2026-04-16

**Authors:** Xiaoqi Wang, Qiaoxi Yang, Jin Bian, Luyun Fan, Xueyan Zhao, Jun Cai

**Affiliations:** 1 Fuwai Hospital, Chinese Academy of Medical Sciences & Peking Union Medical College/National Center for Cardiovascular Diseases, State Key Laboratory of Cardiovascular Disease of China, Beijing, China; 2 Beijing Anzhen Hospital of Capital Medical University and Beijing Institute of Heart Lung and Blood Vessel Diseases, Beijing, China

**Keywords:** CyTOF, cytomegalovirus, hypertension, natural killer cells, single cell RNA sequencing

## Abstract

**Background:**

Innate immune dysregulation is increasingly recognized as a pivotal contributor to hypertension pathogenesis. However, the role of natural killer (NK) cells, a key innate lymphocyte population, remains poorly defined and controversial.

**Method:**

High-dimensional mass cytometry (CyTOF) was employed to profile the innate compartment of peripheral blood mononuclear cells (PBMCs) from 10 hypertensive and 10 normotensive male subjects. A specifically reduced NK subpopulation (CD57^+^CD62L^+^CD161^+^) was identified and subsequently validated in an independent cohort (10 hypertensive and 6 normotensive male subjects) using full-spectrum flow cytometry. The transcriptional heterogeneity and underlying mechanisms of CD57^+^CD62L^+^CD161^+^ NK cells were further delineated by single-cell RNA sequencing.

**Results:**

Innate immune profiling revealed a specific reduction of the CD57^+^CD62L^+^CD161^+^ NK subpopulation in male hypertensive patients, which was confirmed by flow cytometry. Single-cell RNA sequencing of sorted CD57^+^CD62L^+^CD161^+^ NK cells uncovered six transcriptionally distinct subsets and identified a pathogenic shift in their composition. Within the overall diminished pool, hypertension drove a specific depletion of the KLRC2^high^ Adaptive subset while the enhanced cytotoxic, endothelium-interactive FCER1G^high^ Cytotoxic subset was relatively preserved, becoming the dominant population. Mechanistically, the selective loss of the KLRC2^high^ Adaptive subset was associated with impaired IL-15 signaling, which disrupted the balance between pro-survival (*MCL1*, *BCL2*, *PIM2*) and pro-apoptotic (*BCL2L11*, encoding BIM) factors. In contrast, the FCER1G^high^ Cytotoxic subset exhibited relative resistance to this depletion, explaining the observed subset inversion.

**Conclusion:**

Our study demonstrates that hypertension induces a subset-specific remodeling of the human NK cell repertoire, characterized by a global reduction and pathogenic reshaping of the CD57^+^CD62L^+^CD161^+^ NK cells. These findings reveal a novel immune mechanism underlying NK cell dysfunction and vascular inflammation in hypertension.

## 1 Introduction

Hypertension is a major global health burden and a leading risk factor for mortality and disability worldwide (1, 2). Despite its high prevalence, the underlying etiology of hypertension remains incompletely elucidated (3). Growing evidence indicates that hypertension involves a state of chronic low-grade inflammation, underscoring the crucial contribution of the innate immune system to disease initiation and progression (4). As the first line of defense, innate immunity effectively detects danger signals, execute immediate effector functions and initiates inflammatory responses, thereby orchestrating subsequent adaptive immune activation (5). In the context of hypertension, innate immune cells are activated early, releasing pro-inflammatory mediators that promote immune imbalance and vascular dysfunction (6).

Among innate lymphocytes, natural killer (NK) cells have been underexplored in hypertension, despite their established role in vascular homeostasis (7–9). Existing reports on the relationship between NK cells and blood pressure (BP) have yielded conflicting results. NK cells have been implicated in Angiotensin-II induced vascular damage (10), and large-scale immunophenotyping of 1150 human peripheral blood mononuclear cells (PBMCs) has associated a higher proportion of circulating NK cells with elevated systolic BP (11). In contrast, single-cell RNA sequencing on PBMCs (111,694 cells) revealed that the mean proportions of NK cells were significantly lower in hypertensive patients than in controls (12). Moreover, impaired NK cell activity has been correlated with higher BP and an increased incidence of hypertension (13). These inconsistencies may stem from a common methodological limitation: the treatment of NK cells as a homogeneous population, thereby overlooking their well-documented phenotypic and functional heterogeneity.

Human peripheral NK cells are broadly divided into immature CD56^bright^ and mature CD56^dim^ populations, with CD56^dim^ constituting ~90% of circulating NK cells (14). CD57 and L-selectin (CD62L) are established markers essential for delineating the developmental continuum and functional diversity of human NK cells (15). Depending on the surface CD57 and CD62L marker expression, NK cells can be divided into four major subsets that differed not only in the migratory potential to lymphoid (CD62L^+^ NK cells) tissues, but also in the terminal differentiation and cytotoxic capacity due to accumulated cytosolic perforin and granzymes (CD57^+^ NK cells) (16, 17). The expression of CD56^bright^ and CD57 is mutually exclusive in human NK cells, demarcating the immature and mature populations, respectively (18). Critically, within the CD56^dim^ compartment, NK cells exhibit considerable functional heterogeneity, as evidenced by their differential expression of CD57 and CD62L (19).

To resolve this discrepancy in hypertension, we employed high-dimensional mass cytometry (CyTOF) to comprehensively profile the peripheral innate immune compartment in 10 hypertensive and 10 matched normotensive subjects. This approach led to the identification of a specific NK subpopulation—designated NK4—that was significantly reduced in hypertensive patients. This finding was validated in an independent cohort (additional 10 hypertensive and 6 matched normotensive subjects) using full-spectrum flow cytometry. Within this validation cohort, hypertension-diminished NK4 cells were sorted from 5 hypertensive and 5 normotensive subjects for single-cell RNA sequencing to delineate transcriptomic alterations associated with hypertension, thereby providing deeper mechanistic insights into NK cell heterogeneity and functional diversification in this prevalent cardiovascular disease. All participants were male to minimize biological variability and enhance the interpretability, given the well-established sex differences in both BP regulation and immune cell biology.

## 2 Materials and methods

### 2.1 Human samples

**Figure 1:**
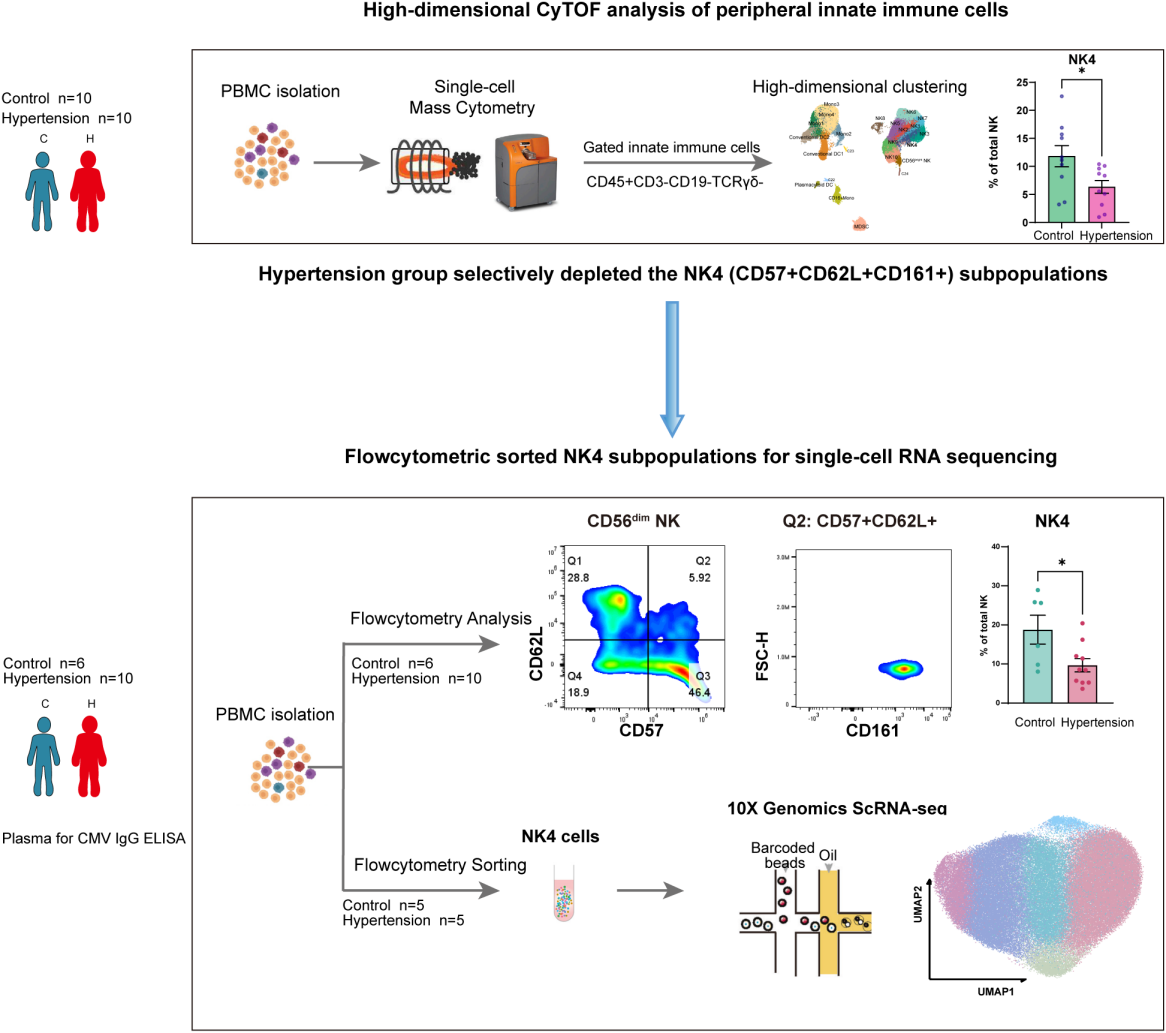
Graphical workflow of integrated multi-omics profiling to identify and characterize a hypertension-diminished NK cell subpopulation. The study design encompasses a discovery phase via high-dimensional mass cytometry (CyTOF), followed by independent validation using full-spectrum flow cytometry, and culminates in mechanistic investigation through single-cell RNA sequencing (scRNA-seq). Discovery Cohort (CyTOF): PBMCs from 10 hypertensive and 10 normotensive subjects were analyzed by CyTOF, leading to the identification of a specific reduced CD57^+^CD62L^+^CD161^+^ NK (originally designated NK4) subpopulation in hypertension. Validation Cohort (Full-Spectrum Flow Cytometry): The reduction of this subpopulation was independently validated in a separate cohort (6 normotensive controls, 10 hypertensive patients; all male) using a Cytek Aurora full−spectrum flow cytometer. Live CD57^+^CD62L^+^CD161^+^ NK cells were sorted for downstream assays. Mechanistic Investigation (scRNA-seq): Sorted CD57^+^CD62L^+^CD161^+^ NK cells from 5 controls and 5 hypertensives underwent scRNA-seq, revealing the transcriptomic heterogeneity and the pathogenic reshaping of the diminished NK4 subpopulation in hypertension. PBMC, peripheral blood mononuclear cell; NK, natural killer. *p < 0.05; standard unpaired Student’s t-test was applied.

Our study was approved by the Ethics Committee of Beijing Fuwai Hospital (Approval No. 2016-838). Informed consents were obtained from all patients and donors prior to enrollment. Participants aged 35–55 years old, including hypertensive patients and normotensive controls matched for age, sex and body mass index were recruited. Peripheral blood samples were collected from two independent cohorts (**Figure 1**): a discovery cohort for CyTOF analysis; an NK-focused validation cohort for full-spectrum flow cytometry and single-cell RNA sequencing. The demographics as described in **Supplementary Tables 1**-**3**, and detailed medication information in **Supplementary Tables 6**-**8**.

Normotensive controls were defined as individuals with no prior history of hypertension and with systolic blood pressure (SBP) < 130 mmHg and diastolic blood pressure (DBP) < 80 mmHg across three consecutive measurements at the time of enrollment. Hypertensive patients were defined as those with a prior diagnosis of hypertension (SBP ≥ 140 mmHg or DBP ≥ 90 mmHg) regardless of antihypertensive medication use. Key exclusion criteria for both groups included: participation in other clinical trials; severe hepatic or renal dysfunction (ALT ≥ 3× upper limit of normal, eGFR < 30 mL/min/1.73 m², or on dialysis); history of stroke, coronary heart disease, or heart failure (NYHA class III/IV); significant valvular, cardiomyopathic, or congenital heart disease; poorly controlled diabetes (fasting blood glucose ≥ 200 mg/dL or HbA1c >8%); severe comorbidities such as malignancy or AIDS with life expectancy <1 year; and any condition deemed by the investigators to preclude safe participation.

### 2.2 Cytometry by time-of-flight

#### 2.2.1 PBMC isolation and preparation

Peripheral blood (5 mL per participant) was collected and processed within 12 hours at room temperature or within 48 hours at 4 °C. All analyses were performed on fresh samples. Peripheral blood mononuclear cells (PBMCs) were isolated by density-gradient centrifugation using Ficoll-Paque PLUS. The cell pellets were washed and resuspended in 5 mL of ice-cold FACS buffer (PBS supplemented with 0.5% BSA), followed by centrifugation at 400 ×g for 5 min at 4 °C. After discarding the supernatant, the cell pellets were resuspended in fresh FACS buffer. Cell count and viability were assessed, and only samples with a minimum of 3×10^6^ cells and viability >85% were used for subsequent staining.

#### 2.2.2 Antibody conjugation and titration

The antibodies and reagents used for mass cytometry are listed in **Supplementary Table 4**. Purified antibodies were purchased from commercial suppliers (BioLegend, eBioscience, BioXcell, R&D Systems, BD Biosciences) and conjugated with appropriate metal isotopes using the MaxPAR Antibody Labeling Kit (Fluidigm) following the manufacturer’s instructions. All conjugated antibodies were titrated to establish optimal staining concentrations before experimental use.

#### 2.2.3 Staining and data acquisition

Cells were washed with PBS and stained with 250 nM cisplatin (Fluidigm) for 5 minutes on ice to discriminate dead cells. After Fc receptor blocking, cells were incubated with a surface antibody cocktail for 30 minutes on ice, washed twice with FACS buffer (1xPBS+0.5%BSA), and fixed overnight in intercalation solution (Maxpar Fix and Perm Buffer containing 250nM 191/193Ir, Fluidigm). For intracellular staining, fixed cells were permeabilized using Perm Buffer (eBioscience), stained with an intracellular antibody cocktail for 30 minutes on ice. Finally, cells were washed and resuspend with deionized water, adding into 20% EQ beads (Fluidigm), acquired on a mass cytometer (Helios, Fluidigm).

#### 2.2.4 Data preprocessing and analysis

Raw data were debarcoded using a doublet-filtering algorithm based on unique mass-tag barcodes (20). FCS files from different batches were normalized using bead-based normalization (21). Data were preprocessed in FlowJo software to remove debris, dead cells, doublets, and normalization beads (**Supplementary Figure 1A**). Following standard data preprocessing, we identified the innate immune compartment (CD3^-^CD19^-^TCRγδ^-^) from live and single CD45^+^ leukocytes for downstream analysis through two-dimensional gating (**Supplementary Figure 1A**). The X-shift algorithm was applied to partition cells into distinct clusters based on marker expression (22). Cell populations were annotated according to canonical marker expression visualized on a heatmap. High-dimensional data were visualized in two dimensions using Uniform Manifold Approximation and Projection (UMAP). Differences in cell population frequencies between groups were assessed using Student’s t-test.

### 2.3 Full-spectrum flow cytometry and cell sorting

For the identification and sorting of NK4 cells from PBMCs, the antibodies are listed in **Supplementary Table 5**. Analysis and sorting were performed on a Cytek Aurora spectral flow cytometer. Prior to sorting, the NK4 population was gated, and a preliminary sample was analyzed to assess cell quality and subset frequency. NK4 cells were subsequently sorted for single-cell RNA sequencing only if the following criteria were met: a minimum of 30,000 cells and post-sort viability exceeding 80%. Flow cytometry data were analyzed using FlowJo software (v10.0), and statistical analyses were performed with GraphPad Prism (v10.0).

### 2.4 Single-cell RNA sequencing and data processing

NK4 cells were isolated by fluorescence-activated cell sorting and processed using the 10x Chromium platform (10x Genomics) to generate single-cell gel bead-in-emulsions (GEMs). Sequencing libraries were constructed according to the manufacturer’s protocol and sequenced on an Illumina NovaSeq 6000 platform. Raw sequencing data (BCL files) were demultiplexed and converted to FASTQ format using Illumina’s bcl2fastq software (v2.20).

Primary data processing was performed using the Cell Ranger pipeline (10x Genomics). Sequencing reads were aligned to the human reference genome (GRCh38), filtered for background noise, and unique molecular identifier (UMI) counts were generated to produce a gene-barcode matrix for each sample. Sample demultiplexing and aggregation were performed using Cell Ranger’s AGGR function to normalize sequencing depths across samples, resulting in a unified gene expression matrix. Finally, after running the whole process, the cell expression matrix including the expression level of genes measured in each cell was loaded into Seurat for downstream analysis.

Prior to downstream analysis, cells were subjected to quality control filtering using the following criteria: (1) number of detected genes per cell >200; (2) mitochondrial gene ratio <25%; and (3) doublet removal using the DoubletFinder package. The downstream Single Cell Data Analysis was performed using the OmicStudio tools created by LC-BIO Co., Ltd (Hangzhou, China) at https://www.omicstudio.cn/cell.

### 2.5 Statistical analysis

Statistical analyses were performed using GraphPad Prism 10.1.2 software. Categorical variables were expressed as counts (percentages), and comparisons between groups were conducted using the two−sided χ² test or Fisher’s exact test, as appropriate. For continuous variables, normality was assessed using the Kolmogorov–Smirnov test. When the sample size was less than 5, the Shapiro–Wilk test was applied instead. Continuous variables that did not follow a normal distribution were presented as median (25th percentile, 75th percentile) and comparisons between two groups were performed using the Mann–Whitney U test. For continuous variables that followed a normal distribution, the F−test was used to assess homogeneity of variances. When variances were unequal, Welch’s corrected unpaired Student’s t−test was applied; when variances were equal, the standard unpaired Student’s t−test was used. A two−sided p−value < 0.05 was considered statistically significant.

## 3 Results

### 3.1 Innate immune profiling reveals a specific reduced NK4 subpopulation in hypertension

**Figure 2:**
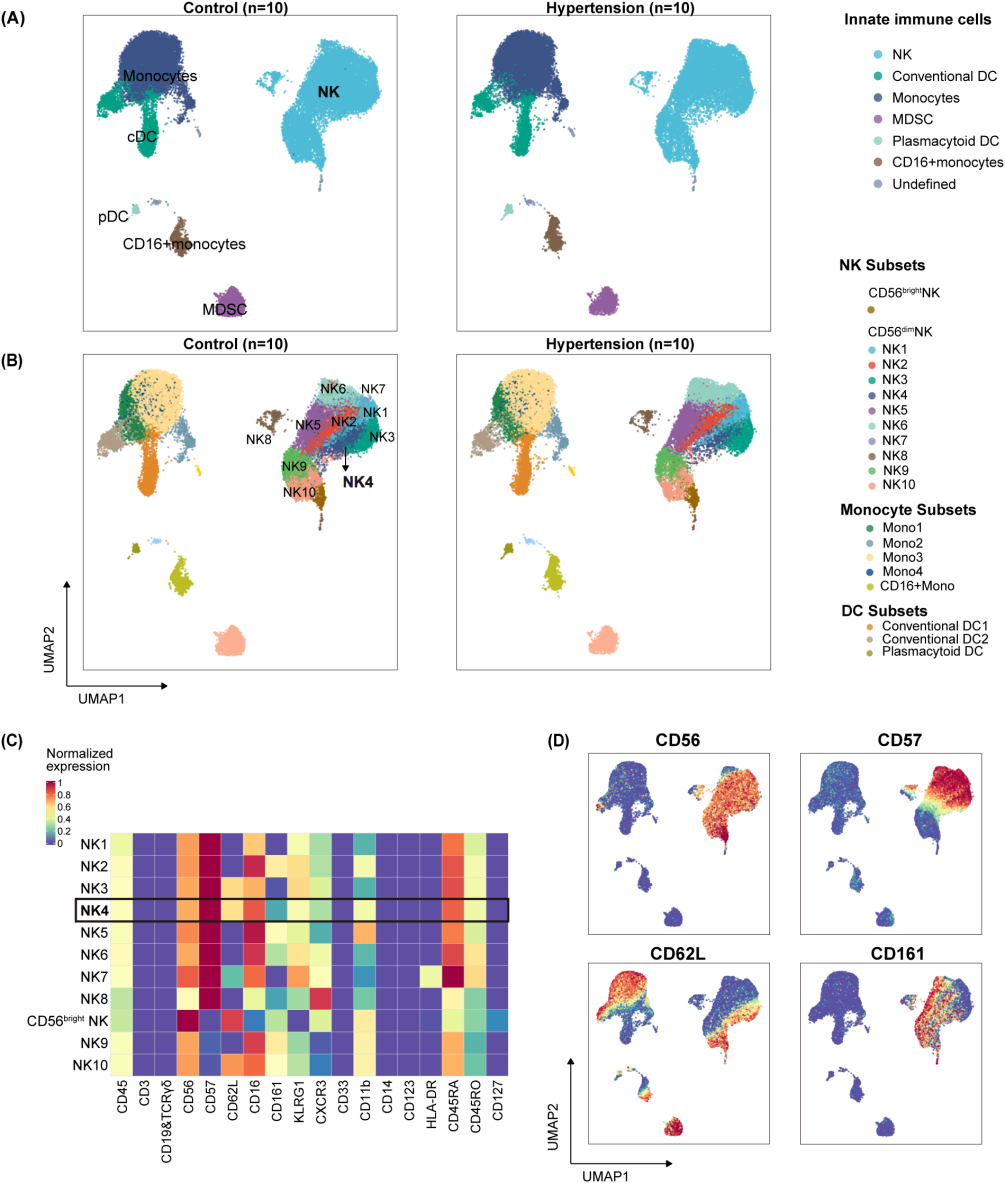
High-dimensional mass cytometry profiling of innate immune compartment in hypertension. Peripheral blood mononuclear cells (PBMCs) were obtained from hypertensive and normotensive control subjects (n = 10 per group). **(A)** UMAP projection of major innate immune cell populations from all 20 subjects. **(B)** UMAP visualization colored by unsupervised clustering revealing innate immune subpopulations. **(C)** Heatmap of normalized marker expression levels for NK cell subpopulations defined in **(B)**. **(D)** Expression of canonical NK cell surface markers overlaid on the UMAP projection from **(B)**. NK, natural killer; Mono, monocytes; DC, Dendritic cell; MDSCs, myeloid-derived suppressor cells.

High-dimensional mass cytometry (CyTOF) profiling of peripheral innate immune cells from 10 hypertensive patients and 10 normotensive controls identified a specific reduction in a natural killer (NK) cell subpopulation in hypertension (**Figure 1**; **Supplementary Table 1**). Following standard data preprocessing (**Supplementary Figure 1A**), unsupervised analysis of the innate immune compartment (CD3^-^CD19^-^TCRγδ^-^) visualized by Uniform Manifold Approximation and Projection (UMAP) delineated major populations, including dendritic cells (DCs), monocytes, myeloid-derived suppressor cells (MDSCs), and NK cells (**Figures 2A, B**). Among these, NK cells exhibited the most pronounced heterogeneity, with the CD56^dim^ compartment segregating into ten distinct subpopulations (NK1–NK10) (**Figure 2B**). The expression patterns of canonical NK cell markers—CD56, CD57, CD62L, CD161 (23)—as visualized in a clustering heatmap (**Figure 2C**) and projected onto UMAP space (**Figure 2D**)—further confirmed the phenotypic and functional diversity of these subpopulations. The detailed immune phenotype of other subsets was visualized in **Supplementary Figures 1B, C**.

**Figure 3:**
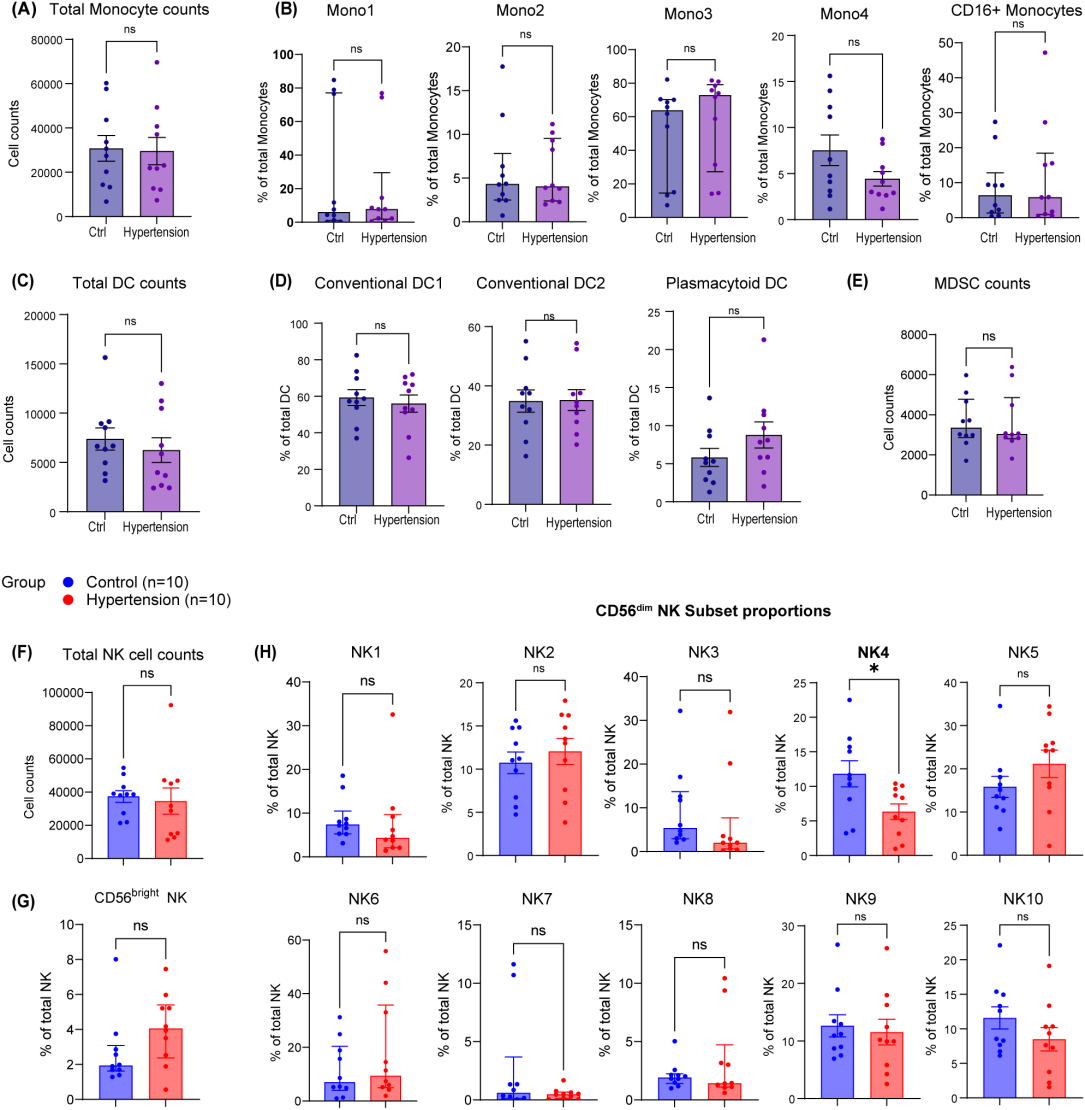
Comparing the subset distribution within each innate immune lineage. Analysis was performed on the CyTOF discovery cohort (10 normotensive controls, 10 hypertensive patients; all male). **(A, B)** Bar plots comparing absolute counts of monocytes **(A)** and frequencies of monocyte subsets within the monocyte lineage **(B)** between hypertensive and control groups. **(C, D)** Bar plots comparing absolute counts of dendritic cells (DCs) **(C)** and frequencies of DC subsets within the DC lineage **(D)** between hypertensive and control groups. **(E)** Bar plot comparing absolute counts of myeloid-derived suppressor cells (MDSCs) between hypertensive and control groups. **(F–H)** Bar plots comparing absolute counts of NK cells **(F)**, and frequencies of CD56^bright^ NK cells **(G)** and CD56^dim^ NK subsets **(H)** within the NK lineage between hypertensive and control groups. Data were assessed for normality using the Kolmogorov−Smirnov test. For comparisons where data were not normally distributed (Mono1, Mono2, Mono3, MDSC count, CD56^bright^ NK, NK1, NK3, NK6, NK7, NK8), the Mann−Whitney U test was used; error bars represent median with 25th and 75th percentiles. For comparisons where data were normally distributed, the F−test was performed to assess variance homogeneity. Total NK counts did not satisfy the assumption of equal variances and were analyzed using Welch’s t−test. All other normally distributed comparisons with equal variances were analyzed using standard unpaired Student’s t−test; bar plots show mean ± SEM. Significance levels: ns, not significant; *p < 0.05. Mono, monocytes; DC, dendritic cells; MDSCs, myeloid−derived suppressor cells; NK, natural killer. Detailed subject characteristics are provided in **Supplementary Tables 1**, **6**, **7**.

While the overall frequencies of major innate immune populations among CD45^+^ leukocytes were comparable between groups (**Supplementary Figures 2A, B**; **Supplementary Data 1**), a pronounced and specific reduction of the NK4 subpopulation was observed in hypertension upon analysis of subset distribution within each lineage [**Figures 3A–H**: (A, B) monocytes; (C, D) DCs; (E) MDSCs; (F -H) NK cells]. This NK4 subpopulation, initially resolved within the CD56^dim^ compartment by CD57 and CD62L co-expression, was unambiguously defined as CD57^+^CD62L^+^CD161^+^ to distinguish it from the phenotypically similar NK3 (also CD57^+^CD62L^+^) subset (**Figures 2C, D**).

The proportion of NK4 subpopulation was significantly lower in hypertensive subjects than in normotensive controls (hypertension vs. control: 6.33% ± 3.59% vs. 11.83% ± 6.00%; p = 0.0231, normally distributed as assessed by the Kolmogorov–Smirnov test, and homogeneity of variances was confirmed using the F-test; a standard unpaired Student’s t-test was applied) (**Supplementary Data 1**). To assess potential confounding by antihypertensive medication, we analyzed medication histories for all hypertensive participants (**Supplementary Table 6**). Stratification by drug class (**Supplementary Table 7**) or number of medications (**Supplementary Table 8**) revealed no consistent association with NK4 subpopulation levels.

This finding provided the rationale for two subsequent steps: independent confirmation of its reduction using full-spectrum flow cytometry in a validation cohort, and its isolation for mechanistic interrogation via single-cell RNA sequencing.

### 3.2 Validation of the NK4 reduction in hypertension by full-spectrum flow cytometry

**Figure 4:**
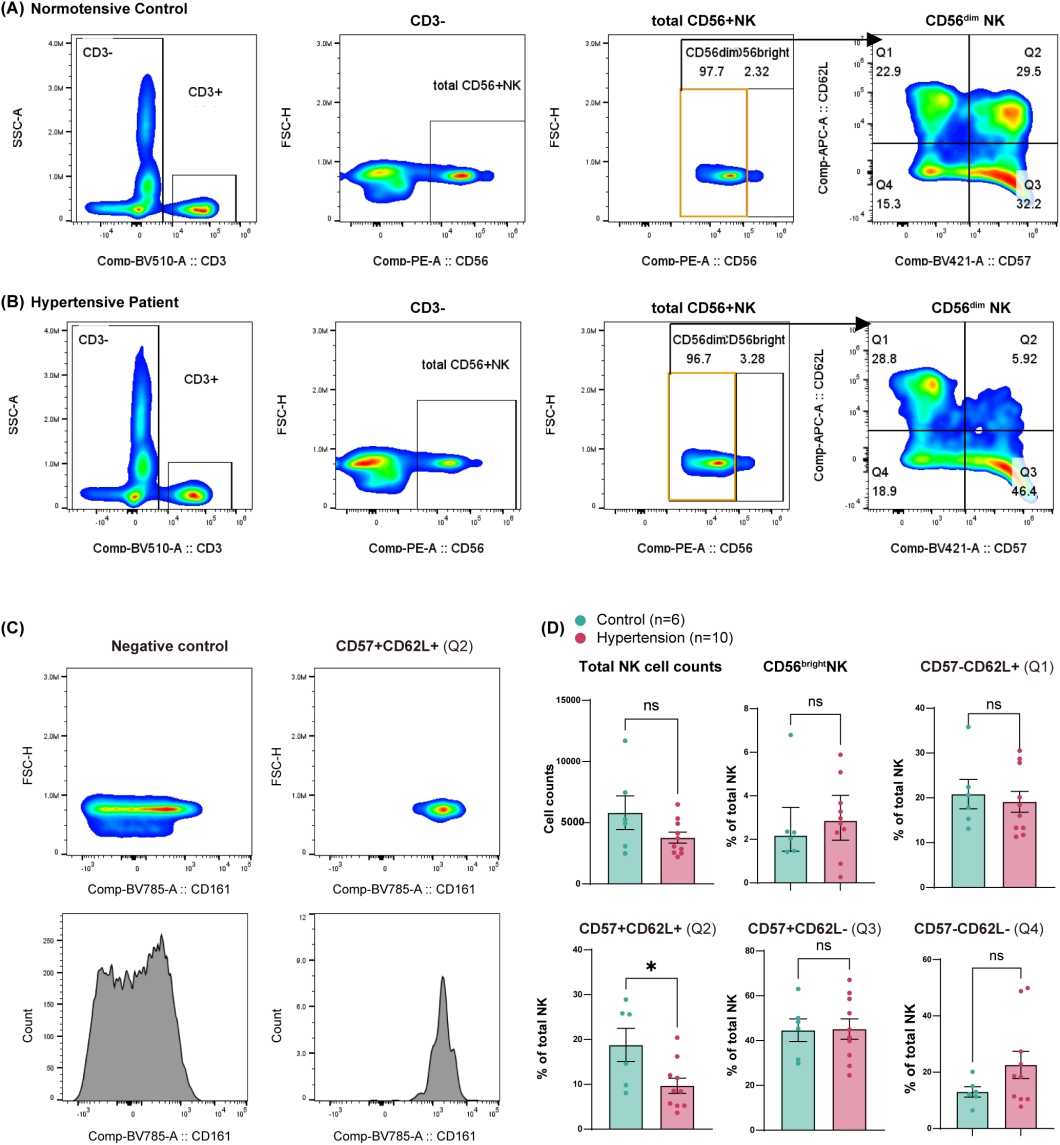
Validation of NK4 subset reduction in hypertension by full-spectrum flow cytometry. Analysis was performed on an independent validation cohort (6 normotensive controls, 10 hypertensive patients; all male) with no overlap to the CyTOF cohort. Data were acquired on a Cytek Aurora full-spectrum flow cytometer and analyzed using FlowJo v10. **(A, B)** Representative gating strategy for the identification of CD57^+^CD62L^+^ NK cells from a normotensive control **(A)** and a hypertensive subject **(B)**. CD3^-^ cells were first gated from live and single cells; then CD3^-^CD56^+^ NK cells were selected; CD56^bright^ and CD56^dim^ NK cells were identified based on CD56 fluorescence intensity. Within the CD56^dim^ compartment, two-dimensional gating based on CD57 and CD62L expression defined four distinct subpopulations. **(C)** Within the CD57^+^CD62L^+^ (Q2) compartment, CD161 expression was confirmed to be uniformly positive, defining the CD57^+^CD62L^+^CD161^+^ NK cell subpopulation. **(D)** Bar plots comparing total NK counts and subset proportions as a percentage of total NK cells between the control (n = 6) and hypertensive (n = 10) groups. Error bars are presented as mean ± SEM. Data were assessed for normality using the Kolmogorov−Smirnov test. For comparisons where data were not normally distributed (CD56^bright^ NK), the Mann−Whitney U test was used; error bars represent median with 25th and 75th percentiles. For comparisons where data were normally distributed, the F−test was performed to assess variance homogeneity. For comparisons with unequal variances (Total NK counts, CD57^-^CD62L^-^ [Q4]), Welch’s t−test was applied. For all other normally distributed comparisons with equal variances, standard unpaired Student’s t−test was used; bar plots show mean ± SEM. Significance levels: ns, not significant; *p < 0.05. Detailed subject characteristics and medication histories are provided in **Supplementary Table 2**, **6**, **7**.

To independently confirm the NK4 reduction observed by CyTOF and to facilitate live-cell sorting for subsequent functional assays, we turned to full-spectrum flow cytometry (**Figure 1**). This analysis was performed on a distinct cohort comprising 6 normotensive controls and 10 hypertensive patients (**Supplementary Table 2**). CD56^+^ NK cells were gated from single, live CD3^-^ cells and further subdivided into CD56^bright^ and CD56^dim^ populations (**Figures 4A, B**). Within the CD56^dim^ compartment, two-dimensional gating based on CD57 and CD62L expression defined four distinct subpopulations (**Figures 4A, B**). The CD57^+^CD62L^+^ population was uniformly positive for CD161, corresponding to the phenotype of the NK4 subpopulation as CD57^+^CD62L^+^CD161^+^ (**Figure 4C**). Comparing the proportions of these NK subpopulations between normotensive (n=6) and hypertensive (n=10) subjects revealed a significant reduction specifically in the CD57^+^CD62L^+^CD161^+^ NK cells in hypertension (hypertension vs. control: 9.68% ± 5.38% vs. 18.80% ± 9.07%; p = 0.0232; normally distributed with homogeneous variances, a standard unpaired Student’s t-test was applied) (**Figure 4D**; **Supplementary Data 2**).

To address the concern regarding potential unmixing or compensation artifacts in the bivariate plots of CD62L (X-axis) versus CD57 (Y-axis), we applied an alternative gating strategy to independently verify the CD57^+^CD62L^+^ population (**Supplementary Figures 3A, B**). Specifically, CD56^dim^ NK cells were first gated, and the CD56^dim^CD57^+^ subset was subsequently selected using CD57 (X-axis) versus CD56 (Y-axis). From this CD56^dim^CD57^+^ gate, we then identified the CD57^+^CD62L^+^ population, which was also uniformly positive for CD161 (**Supplementary Figure 3C**). Using this alternative gating strategy, we compared the proportions of CD57^+^CD62L^+^ NK cells between normotensive (n=6) and hypertensive (n=10) subjects. A significant reduction was observed in hypertension (hypertension vs. control: 10.73% ± 5.44% vs. 20.74% ± 9.38%; p = 0.016; normally distributed with homogeneous variances, a standard unpaired Student’s t-test was applied), consistent with our original finding (**Supplementary Figure 3D**). This result further supports that the observed CD57^+^CD62L^+^CD161^+^ NK subpopulation is biologically genuine and not attributable to unmixing or compensation artifacts.

In this validation cohort, we also analyzed medication histories (**Supplementary Table 6**) stratified by drug class (**Supplementary Table 7**) or number of medications (**Supplementary Table 8**). The results revealed no consistent association with the proportions of CD57^+^CD62L^+^CD161^+^ NK cells. Given the established influence of cytomegalovirus (CMV) infection on NK cell repertoire (24), we performed a sensitivity analysis by excluding the two CMV IgG^-^ hypertensive subjects, thereby retaining all CMV IgG^+^ subjects for comparative analysis. The reduction in CD57^+^CD62L^+^CD161^+^ NK cells remained statistically significant after this adjustment (hypertension vs. control: 10.15% ± 5.57% vs. 18.80% ± 9.07%; p = 0.047; normally distributed with homogeneous variances, a standard unpaired Student’s t-test was applied), confirming that its depletion is a specific feature of hypertension (**Supplementary Figures 4A, B**; **Supplementary Data 2**).

### 3.3 Single-cell RNA sequencing reveals transcriptional heterogeneity within the hypertension-diminished NK4 cells

**Figure 5:**
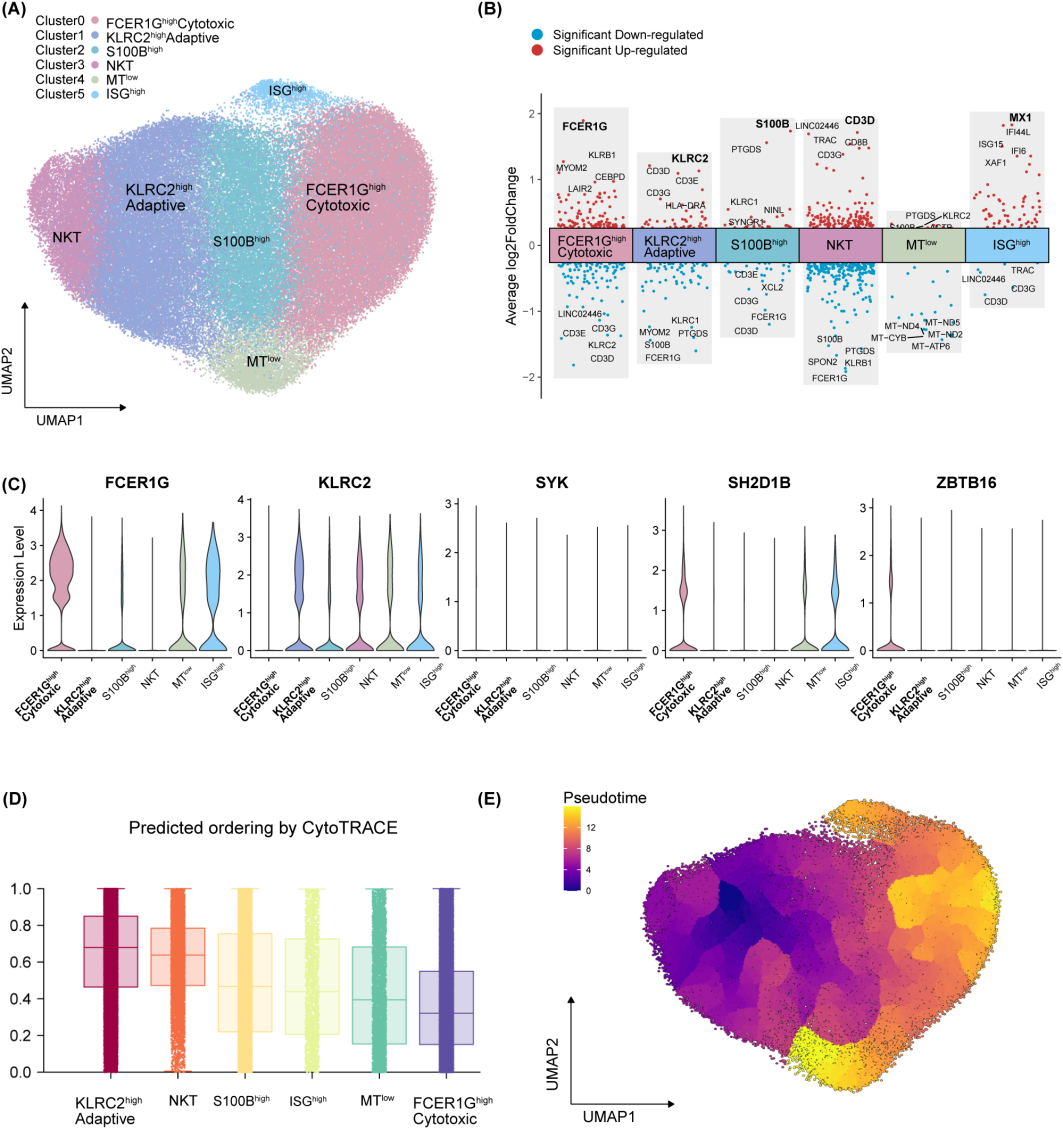
Transcriptional heterogeneity in flowcytometric sorted hypertension-diminished NK4 cells. **(A)** UMAP showing unsupervised clustering of 94739 high-quality NK4 cells. **(B)** Volcano plot displaying the top five up-regulated and down-regulated marker genes of each subset identified in **(A)**. **(C)** Violin plot depicting the transcription levels of crucial genes used to discriminate the adaptive NK subset from conventional NK cells, including *FCER1G*, *KLRC2*, *SYK*, *SH2D1B* and *ZBTB17*. **(D)** Predicted ordering by CytoTRACE analysis. Cells with high scores indicate the less mature state, higher level of developmental potential and transcriptional diversity. **(E)** Monocle3 pseudotime inference from a starting point as KLRC2^high^ Adaptive subset based on the CytoTRACE analysis. The scale indicates the maturation state, from dark purple (least mature) to yellow (most mature).

To delineate the transcriptomic landscape of the hypertension-diminished NK4 population, we sorted these cells from 5 normotensive and 5 hypertensive subjects from the validation cohort for 10x Genomics single-cell RNA sequencing (**Figure 1**; **Supplementary Table 3**). After stringent quality control (**Supplementary Data 3**), 94,739 high-quality single-cell transcriptomes were retained for unsupervised clustering, which partitioned the NK4 cells into six distinct transcriptional states (**Figure 5A**). We annotated these clusters based on their discriminative marker genes (**Figure 5B**; **Supplementary Data 3**), a nomenclature strategy consistent with the convention of defining subsets by high-fidelity markers (25).

Cluster 0 was designated as the FCER1G^high^ Cytotoxic subset, characterized by high expression of *FCER1G* (**Figures 5B, C**; **Supplementary Data 3**), a common immunoreceptor signaling adaptor (26) and a marker of conventional innate NK cells to discriminate from adaptive NK cells (27). The feature genes of Cluster 0 (**Supplementary Data 3**) also included a suite of cytotoxic effector molecules including *PRF1*, *GNLY*, *GZMA*, and *GZMM*; as well as components of the IL-2/IL-15 signaling pathway, including *JAK1* and *IL2RB* (28). This profile signifies a mature and highly active cytotoxic phenotype (29). In contrast, Cluster 1 was annotated as the KLRC2^high^ Adaptive subset, reflecting a transcriptional profile typical of adaptive NK cells (**Figures 5B, C**; **Supplementary Data 3**). Unlike conventional innate NK cells, adaptive NK cells—often expanded in response to CMV infection—can exhibit antigen-specific clonal expansion and long-term memory (30). Key adaptive characteristics of Cluster 1 (**Figures 5B, C**) included high expression of *KLRC2* (encoding NKG2C) and marked downregulation of *FCER1G* (31), along with reduced expression of signaling mediators (*SYK*, *SH2D1B*) and the transcription factor *ZBTB16* (32). These results underscoring a functional dichotomy between the FCER1G^high^ Cytotoxic and KLRC2^high^ Adaptive subsets.

The remaining clusters were defined by distinct transcriptional programs: Cluster 2 (S100B^high^) by upregulated *S100B*. Cluster 3 (NKT-like) showed expression of T-cell receptor (TCR) and coreceptor genes (*TRAC*, *CD3D*, *CD3G*, *CD8B*). Notably, these cells were sorted from the CD3^-^CD56^+^ gate. This observation may reflect a population of activated NKT cells that have low/absent surface CD3 protein expression, as previous studies have proved that activated NKT cells downregulate surface TCR expression by flow cytometry (33). We therefore refer to this cluster descriptively as the “NKT-like”. Cluster 4 (MT^low^) by downregulation of mitochondrial (MT) genes; and Cluster 5 (ISG^high^) by a pronounced interferon-stimulated gene (ISG) signature (**Figure 5B**; **Supplementary Data 3**). Developmental trajectory analysis using CytoTRACE (34) predicted the highest differentiation potential in the KLRC2^high^ Adaptive subset and the lowest in the FCER1G^high^ Cytotoxic subset (**Figure 5D**). The KLRC2^high^ Adaptive subset with the highest CytoTRACE score mapped to the starting point of the trajectory by applying the pseudotime algorithm Monocle3 (**Figure 5E**). The most mature developmental stage of the FCER1G^high^ Cytotoxic subset supported their superior cytotoxic activity, since progression of NK cell maturation involves the acquisition of effector molecules and function (35). Collectively, these results uncover extensive transcriptional heterogeneity within the hypertension-diminished NK4 population, revealing distinct cytotoxic, adaptive, and specialized subsets.

### 3.4 Hypertension drives a pathogenic inversion within the NK4 compartment

**Figure 6:**
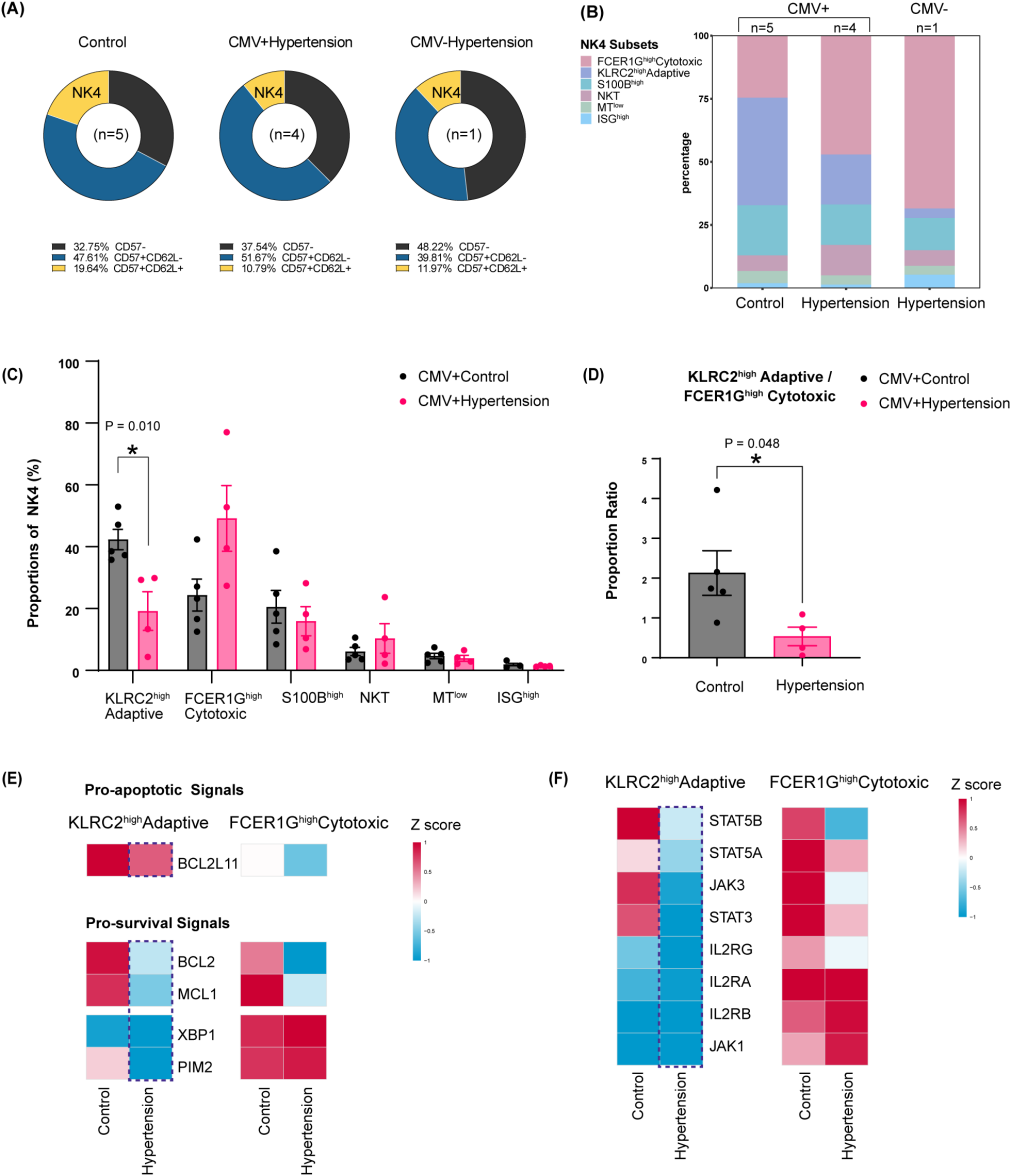
Pathogenic inversion within the CD57^+^CD62L^+^CD161^+^ NK cells in hypertension. Analysis was performed on sorted CD57^+^CD62L^+^CD161^+^ NK cells from the scRNA-seq cohort. To control for CMV-driven effects, subsequent analyses focused on CMV+ individuals (5 normotensive controls, 4 hypertensive patients; all male, age-matched). Data were acquired on a Cytek Aurora full-spectrum flow cytometer for sorting, and scRNA-seq was performed using the 10x Genomics platform. **(A, B)** Data are presented for 5 CMV IgG (+) normotensive controls, 4 CMV IgG (+) hypertensive patients, and 1 CMV IgG (-) hypertensive patient. **(A)** Spectral flow cytometry data showing CD57^+^CD62L^+^CD161^+^ NK cells as a percentage of total CD56^+^ NK cells. **(B)** Stacked bar plot showing the proportional composition of the six transcriptionally distinct subsets within the CD57^+^CD62L^+^CD161^+^ NK cells. **(C)** Proportion comparison of each NK4 subset between CMV+ control (n=5) and CMV+ hypertension (n=4). **(D)** Comparison of the KLRC2^high^/FCER1G^high^ subset proportion ratio between CMV+ controls and CMV+ hypertensive patients. **(C, D)** Data were assessed for normality using the Shapiro−Wilk test and were found to be normally distributed; homogeneity of variances was confirmed using the F−test. Statistical significance was determined by standard unpaired Student’s t−test. Error bars represent mean ± SEM. *p < 0.05. **(E)** Heatmap depicting expression levels of crucial pro-survival/anti-apoptotic molecules (*MCL1*, *BCL2*, *XBP1* and *PIM2*) and the pro-apoptotic factor *BCL2L11* (encoding BIM) in the KLRC2^high^ Adaptive subset and FCER1G^high^ Cytotoxic subset. **(F)** Heatmap depicting the expression levels of crucial molecules in IL15/JAK/STAT pathway in the KLRC2^high^ Adaptive subset and FCER1G^high^ Cytotoxic subset. For both heatmaps, rows are scaled by Z-score, and expression levels are derived from scRNA-seq data of sorted CD57^+^CD62L^+^CD161^+^ NK cells (5 CMV+ controls and 4 CMV+ hypertensives). Abbreviations: CMV, cytomegalovirus; KLRC2, killer cell lectin−like receptor C2 (NKG2C); FCER1G, Fc epsilon receptor Ig; IL−15, interleukin−15; JAK, Janus kinase; STAT, signal transducer and activator of transcription.

Single-cell RNA sequencing of NK4 cells from10 subjects, comprising 5 normotensive controls and 5 hypertensive patients (**Figure 6A**), revealed a profound shift in subset composition within this compartment (**Figure 6B**). To control for the cytomegalovirus (CMV)-driven effects, subsequent analysis focused on 9 CMV IgG^+^ individuals (5 controls, 4 hypertensive) (**Figures 6C–F**). Within the NK4 compartment, the KLRC2^high^ Adaptive subset was the predominant population in normotensive controls (mean ± SD: 42.35% ± 7.39%) but was significantly depleted in hypertension (19.22% ± 12.48%; p = 0.010; normally distributed as assessed by the Shapiro–Wilk test, and homogeneity of variances was confirmed using the F-test, a standard unpaired Student’s t-test was applied) (**Figure 6C**; **Supplementary Data 3**). In parallel, the FCER1G^high^ Cytotoxic subset showed an increasing, though not statistically significant, trend in hypertension (control vs. hypertension: 24.38 ± 11.54% vs. 49.18 ± 21.27%; p = 0.059; normally distributed with homogeneous variances, a standard unpaired Student’s t-test was applied) (**Figure 6C**; **Supplementary Data 3**). This cellular redistribution resulted in a substantial inversion of the subset ratio, with the KLRC2^high^/FCER1G^high^ proportion ratio dropping from 2.13 ± 1.25 in control to 0.54 ± 0.47 in hypertension (p = 0.048; normally distributed with homogeneous variances, a standard unpaired Student’s t-test was applied) (**Figure 6D**; **Supplementary Data 3**).

To decipher the functional implications of this inversion in hypertension, we compared differentially expressed genes (DEGs) between the two key subsets in CMV^+^ hypertensive individuals (**Supplementary Data 3**). KEGG pathway analysis of genes upregulated in the FCER1G^high^ Cytotoxic subset revealed significant enrichment of “NK cell–mediated cytotoxicity” and “Leukocyte trans-endothelial migration” (**Supplementary Figure 5A**). The FCER1G^high^ Cytotoxic subset demonstrated a markedly amplified cytotoxic signature, characterized by the coordinated upregulation of pivotal effector molecules. These included *PRF1* and *GZMB*—core genes previously linked to blood pressure regulation in large-scale transcriptomic studies (36) —as well as *GZMA* and *GZMM*, the death receptor ligand *FASLG* (37), and *FCGR3A* (encoding CD16), the pivotal receptor for antibody-dependent cellular cytotoxicity (ADCC) (38) (**Supplementary Data 3**; **Supplementary Figure 5B**). Complementing this, the subset also exhibited a profile primed for vascular engagement, characterized by the expression of adhesion (*ITGAM*, *ITGB2*), rolling (*SELL*, *SELPLG*), and signaling (*CD226*) (**Supplementary Data 3**; **Supplementary Figure 5C**). Collectively, this transcriptional program enables the efficient recruitment and effector function of the FCER1G^high^ Cytotoxic subset at sites of activated endothelium, a hallmark of hypertensive vascular beds.

Thus, within the context of a shrinking NK4 pool, hypertension specifically depletes the KLRC2^high^ Adaptive subset while promoting a relative expansion of a cytotoxic, vasculature-interacting subset. This reprogramming of the NK4 compartment likely constitutes a key mechanism fueling chronic vascular inflammation and injury in hypertension.

### 3.5 Impaired IL-15 signaling underlies the selective loss of the KLRC2^high^ adaptive subset in hypertension

Given the specific reduction of the KLRC2^high^ Adaptive subset, we hypothesized a disruption in cellular survival homeostasis (39). We therefore analyzed key NK-cell survival and apoptotic regulators, focusing on the BCL-2 protein family where pro-survival members (MCL1, BCL2) counteract the pro-apoptotic effects of BIM, with MCL1 serving as the dominant survival factor (40, 41). The KLRC2^high^ Adaptive subset, the most predominant component within NK4 cells in normotensive controls, maintained a high-level equilibrium, co-expressing the pro-survival signals (*MCL1*, *BCL2*) and the pro-apoptotic factor (*BCL2L11*, encoding BIM) (**Figure 6E**). This equilibrium was severely skewed in hypertension. The KLRC2^high^ Adaptive subset from hypertensive individuals displayed coordinated downregulation of *MCL1* and *BCL2*, while *BCL2L11* expression remained high, creating a potent pro-apoptotic bias (**Figure 6E**). Concurrently, expression of *PIM2*, a critical kinase in the XBP1-PIM2 survival pathway (42), was reduced. As these NK survival systems are regulated by IL-15 signaling via JAK/STAT pathways (40, 43), we profiled this axis and found a broad downregulation of core components in the hypertensive KLRC2^high^ Adaptive subset, including *STAT5B*, *STAT5A*, *STAT3*, and *JAK3* (**Figure 6F**).

Conversely, the FCER1G^high^ Cytotoxic subset in hypertension exhibited a different pattern: although *MCL1* and *BCL2* were lower, *BCL2L11* was also downregulated, and the XBP1-PIM2 axis remained active (**Figure 6E**). This divergent survival signaling indicates that the FCER1G^high^ cytotoxic subset is relatively resistant to this depletion, clarifying the subset inversion. Thus, hypertension selectively disables the survival machinery in the KLRC2^high^ Adaptive subset via defective IL-15 signaling, disrupting the MCL1/BCL2–BIM balance and the XBP1-PIM2 axis, and leading to its preferential demise.

## 4 Discussion

In this study, we unveil a previously unrecognized abnormality in the innate immune landscape of human hypertension: the specific reduction and transcriptional reprogramming of CD57^+^CD62L^+^CD161^+^ NK cells. By integrating CyTOF, flow cytometry, and scRNA-seq, we demonstrate that hypertension is not merely associated with a global decline in CD57^+^CD62L^+^CD161^+^ NK cells, but drives a fundamental inversion of its constituent subsets, shifting the balance from a dominant adaptive state towards a pro-inflammatory cytotoxic one. This finding deepens our understanding of immune dysregulation in hypertension beyond the established roles of T cells and monocytes.

Contradictory reports regarding the association between NK cells and blood pressure regulation have long presented a challenge in the field (11, 13). Two earlier studies also utilized CyTOF for deep immunophenotyping of PBMCs but did not identify significant changes in NK cell compartments between hypertensive and control groups (44, 45). One reported no alteration in total NK cells as a percentage of CD45^+^ leukocytes (44), which aligns with our observation. The other study by Yang et al. (45) classified NK cells into four broad subsets; however, their antibody panel did not include CD62L, a key marker required to identify the hypertension-diminished CD57^+^CD62L^+^CD161^+^ NK cells. Another key distinction lies in the age distribution of the study cohorts. The mean age in Yang et al.’s cohort (45) (23.2 ± 0.8 years for control; 35.2 ± 4.9 years for hypertension) is considerably lower than that in our CyTOF (48.50 ± 5.76 vs. 50.40 ± 3.27 years) and flow cytometry (44.00 ± 7.87 vs. 40.60 ± 2.99 years) cohorts, which may further explain the divergent findings regarding NK subset dynamics.

Our initial discovery, enabled by high-dimensional CyTOF and independently validated, was the specific depletion of the CD57^+^CD62L^+^CD161^+^ NK cells (NK4 subpopulation) in hypertension. However, regarding the resolution of NK cell subpopulations, we acknowledge that the continuous expression gradient of CD161 and the limited resolution of full-spectrum flow cytometry prevented the identification of the NK3 (CD161^-^) subset as originally defined by CyTOF. The near-uniform positivity of CD161 within the CD57^+^CD62L^+^ gate suggests that our flow cytometric sorting and validation predominantly captured the CD57^+^CD62L^+^CD161^+^ NK cells. The reproducible reduction of this population across two independent cohorts using two distinct technologies (CyTOF and flow cytometry) reinforces the robustness of our primary finding, despite the inherent limitations of subset resolution.

A recent report (17) also indicated that the maintenance of this specialized lymphocyte pool is compromised in the chronic disease status. Patients with post-COVID syndrome also showed depletion in CD57^+^CD62L^+^ NK cells compared with control (p = 0.009) (17). We therefore posit that the chronic, low-grade inflammatory milieu of hypertension may similarly deplete NK4. Our results help reconcile prior contradictions by demonstrating that hypertension remodels the human NK cell repertoire at a subset-specific level.

We acknowledge that the informative analyses about antihypertensive treatment, cannot completely exclude the possibility of subtle modulatory effects of specific medications on NK cell biology. The ideal study design to definitively address this question would be a prospective longitudinal study enrolling newly diagnosed, treatment-naïve hypertensive patients with sampling before and after initiation of standardized therapy.

The scRNA-seq data subsequently revealed that this phenotypically uniform population is, in fact, a composite of multiple transcriptional states. The most dramatic change was the specific collapse of the KLRC2^high^ Adaptive subset, a population sharing key features with adaptive NK cells known to expand in response to CMV. The concurrent relative preservation of the highly cytotoxic FCER1G^high^ subset creates a new cellular equilibrium within the diminished NK4 pool. This inversion of the KLRC2^high^/FCER1G^high^ ratio is a central finding, suggesting that the quality of the remaining NK cell response is as important as its overall magnitude. The functional profile of the expanding FCER1G^high^ Cytotoxic subset in hypertension is particularly suggestive of a direct role in vascular injury. This subset is molecularly primed for both potent cytotoxicity, via high expression of *PRF1*, *GZMB*, *FCGR3A* and *FASLG*, and efficient recruitment to activated endothelium, via upregulated integrins and homing receptors. Given that hypertensive vasculature is characterized by endothelial activation with increased adhesion molecule expression (6), this subset is ideally positioned to engage with and inflict damage upon the vessel wall. This aligns with human genetic evidence linking cytotoxic molecules like *GZMB* and *PRF1* to blood pressure regulation (36), providing a plausible mechanistic bridge between our observed NK subset shift and end-organ damage.

While our transcriptomic data predict a heightened cytotoxic and vasculature−interactive capacity of the FCER1G^high^ Cytotoxic subset, direct functional validation is required to establish its contribution to vascular injury. Future studies should assess degranulation, target cell killing, endothelial adhesion, and trans−endothelial migration using isolated NK cell subsets from hypertensive and normotensive individuals. Such functional assays will be essential to translate these findings into a mechanistic understanding of NK cell−mediated vascular damage in hypertension.

A key mechanistic insight of our work is the elucidation of why the KLRC2^high^ Adaptive subset is selectively vulnerable. We identified a defect in the core IL-15 signaling pathway, a non-redundant survival signal for NK cells (40, 43), specifically within this subset in hypertensive subjects. The downstream consequence is a fatal imbalance in the BCL-2 family regulation, with loss of pro-survival *MCL1* and *BCL2* while pro-apoptotic *BCL2L11* (encoding BIM) remains high. In contrast, the FCER1G^high^ Cytotoxic subset, which co-downregulated *BCL2L11* and maintained *PIM2* expression, was more resilient. This demonstrates a subset-specific susceptibility to the hypertensive milieu, ultimately leading to the preferential demise of adaptive NK subset. While our data implicate impaired IL-15 signaling as a key driver of KLRC2^high^ Adaptive subset depletion in hypertension, the precise origin of this defect remains to be fully elucidated. The coordinated downregulation of STAT5A, STAT5B, and JAK3 points to a disruption within the downstream signaling cascade, but whether this is triggered by receptor downregulation or intrinsic signaling desensitization requires further investigation. Moreover, *in vitro* experiments are necessary to determine whether the observed defects are reversible after treatment with IL-15 agonists. Addressing these questions could open new therapeutic avenues for mitigating the immune dysregulation in hypertension.

We recognize that the sample sizes in this study, particularly for the scRNA-seq analysis after CMV stratification, are limited and may not fully capture the heterogeneity of the hypertensive population. Future studies with larger, well-powered cohorts are necessary to confirm these observations, to dissect the interaction between CMV serostatus and hypertension, and to rule out confounding by individual variation. Another key limitation of this study is the predominantly CMV IgG (+) composition of our cohorts; these findings cannot be generalized to CMV IgG (-) hypertensive patients. Future studies should include well-powered cohorts of both CMV IgG (+) and CMV IgG (-) individuals to determine whether the observed NK cell alterations are modified by CMV status.

In conclusion, our multi-omics approach reveals that hypertension remodels the human NK cell repertoire at a subset-specific level. The disease does not simply cause a generalized deficiency but actively reprograms the NK4 compartment. This involves the selective ablation of the KLRC2^high^ Adaptive subset associated with IL-15 signaling failure, coupled with a relative predominance of a cytotoxic, endothelium-interactive subset. These findings provide a critical, previously unknown mechanism driving the immune dysregulation in hypertension. Therapeutic strategies aimed at rebalancing NK cell subset homeostasis may therefore offer novel avenues for immunomodulatory interventions in cardiovascular disease.

## Data Availability

The Single-cell RNA sequence data presented in the study are deposited in the Genome Sequence Archive (Genomics, Proteomics & Bioinformatics 2025) repository, accession number (GSA-Human: HRA014291).
