# Supplementary material for: Innate immune profiling reveals a specific reduction of CD57+CD62L+CD161+ NK cells in CMV-positive males with hypertension
Source: Front Immunol. 2026 Apr 16;17:1749702. doi: 10.3389/fimmu.2026.1749702 (PMC13128649; doi:10.3389/fimmu.2026.1749702)
Supplement: Supplementary file 1 [file DataSheet1.zip › revised Supplementary Figures and legend/Supplementary Figure legend.docx]

Supplementary Material

# Supplementary Figures

## Supplementary Figure 1

**Supplementary Figure 1.** **High-dimensional mass cytometry profiling of innate immune cells in hypertension.** Peripheral blood mononuclear cells (PBMCs) were obtained from hypertensive and normotensive control subjects (n = 10 per group). **(A).** Standard data preprocessing (remove debris, dead cells, doublets, and normalization beads) to gate live, single CD45⁺ leukocytes, identified the innate immune compartment (CD3⁻CD19⁻TCRγδ⁻) from for downstream analysis. **(B).** Expression of canonical monocyte and DC cell surface markers overlaid on the UMAP projection from Figure 2A. **(C).** Heatmap of normalized marker expression levels for monocyte and DC cell subpopulations defined in Figure 2B.

## Supplementary Figure 2

**Supplementary Figure 2.** **Comparing the frequencies of major innate immune populations (A)** Bar plots with overlaid data points comparing absolute counts of CD45⁺ leukocytes between hypertensive and control groups (n = 10 per group). **(B)** Comparing the frequencies of major innate immune populations among CD45⁺ leukocytes. Abbreviations: NK: natural killer; DC: Dendritic cell; MDSCs: myeloid-derived suppressor cells. Error bars are presented as mean ± SEM. Statistical significance was determined by two-tailed unpaired t-tests; ns, not significant; p < 0.05 was considered significant.

## Supplementary Figure 3

**Supplementary Figure 3. Independent verification of the CD57⁺CD62L⁺ population using an alternative gating strategy to exclude unmixing or compensation artifacts.** Analysis was performed on an independent validation cohort (6 normotensive controls, 10 hypertensive patients; all male) with no overlap to the CyTOF cohort. Data were acquired on a Cytek Aurora full‑spectrum flow cytometer and analyzed using FlowJo v10. **(A, B)** Sequential gating strategy for identification of CD57⁺CD62L⁺ NK cells from a normotensive control **(A)** and a hypertensive subject **(B)**. CD56ᵈⁱᵐ NK cells were first gated from live, single CD3⁻CD56⁺ NK cells. The CD56ᵈⁱᵐCD57⁺ subset was then selected using CD57 (X‑axis) versus CD56 (Y‑axis). From this CD56ᵈⁱᵐCD57⁺ gate, the CD57⁺CD62L⁺ population was identified. **(C)** Within the CD57⁺CD62L⁺ population, CD161 expression was confirmed to be uniformly positive, defining the CD57⁺CD62L⁺CD161⁺ NK cell subpopulation. **(D)** Bar plots comparing CD57⁺CD62L⁺CD161⁺ NK proportions as a percentage of total NK cells between the control (n = 6) and hypertensive (n = 10) groups. Error bars are presented as mean ± SEM. Statistical significance was determined using an unpaired Student’s t‑test after confirmation of normality (Kolmogorov–Smirnov test) and homogeneity of variances (F‑test). Error bars are presented as mean ± SEM; p < 0.05 was considered significant; *p <0.05.

## Supplementary Figure 4

**Supplementary Figure 4.** **Validation of CD57⁺CD62L⁺CD161⁺ NK cell reduction in hypertension by full-spectrum flow cytometry with CMV stratification.** Analysis was performed on the validation cohort (6 CMV+ normotensive controls, 8 CMV+ hypertensive patients and 2 CMV- hypertensive patients; all male). Data were acquired on a Cytek Aurora full‑spectrum flow cytometer and analyzed using FlowJo v10. **(A)** CD57⁺CD62L⁺CD161⁺ NK cells as a percentage of total CD56⁺ NK cells. Data are presented for 6 CMV+ normotensive controls, 8 CMV+ hypertensive patients, and 2 CMV- hypertensive patients. **(B)** Bar plot comparing total NK cell counts between CMV⁺ controls (n = 6) and CMV⁺ hypertensive patients (n = 8). **(C, D)** Bar plots comparing frequencies of CD56ᵇʳⁱᵍʰᵗ NK cells (C) and CD56ᵈⁱᵐ NK subsets (D) within the NK cell lineage between CMV+ controls (n = 6) and CMV+ hypertensive patients (n = 8). Data were assessed for normality using the Kolmogorov‑Smirnov test. For comparisons where data were not normally distributed (CD56ᵇʳⁱᵍʰᵗ NK), the Mann‑Whitney U test was used; error bars represent median with 25th and 75th percentiles. For comparisons where data were normally distributed, the F‑test was performed to assess variance homogeneity. For the comparison with unequal variances (CD57⁻CD62L⁻ [Q4]), Welch’s t‑test was applied. For all other normally distributed comparisons with equal variances, standard unpaired Student’s t‑test was used; bar plots show mean ± SEM. Significance levels: ns, not significant; *p < 0.05.

## Supplementary Figure 5

**Supplementary Figure 4.** **KEGG analysis of differentially expressed genes (DEGs) upregulated in the FCER1G^high^ Cytotoxic subset versus the KLRC2^high^ Adaptive subset.** **(A)** KEGG pathway analysis revealed significant enrichment of “NK cell–mediated cytotoxicity” and “Leukocyte trans-endothelial migration”. **(B)** The red mark represented the DEGs enriched in “NK cell–mediated cytotoxicity” pathway. **(C)** The red mark represented the DEGs enriched in “Leukocyte trans-endothelial migration”.
