# Supplementary figures and images for: Innate immune profiling reveals a specific reduction of CD57+CD62L+CD161+ NK cells in CMV-positive males with hypertension
Source: Front Immunol. 2026 Apr 16;17:1749702. doi: 10.3389/fimmu.2026.1749702 (PMC13128649; doi:10.3389/fimmu.2026.1749702)

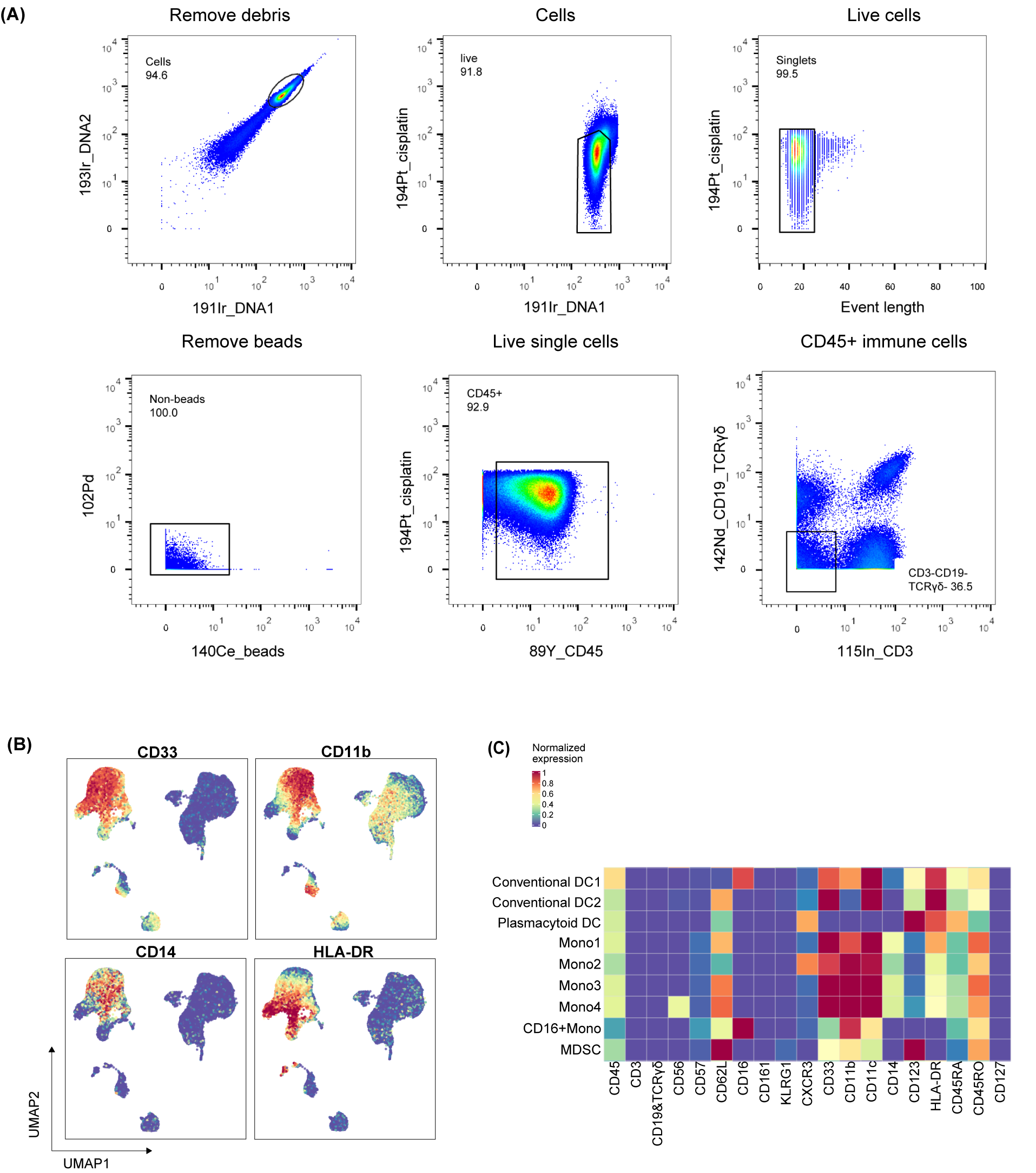

Supplement: Supplementary file 1 [file DataSheet1.zip › revised Supplementary Figures and legend/Supplementary Figure 1.tif]

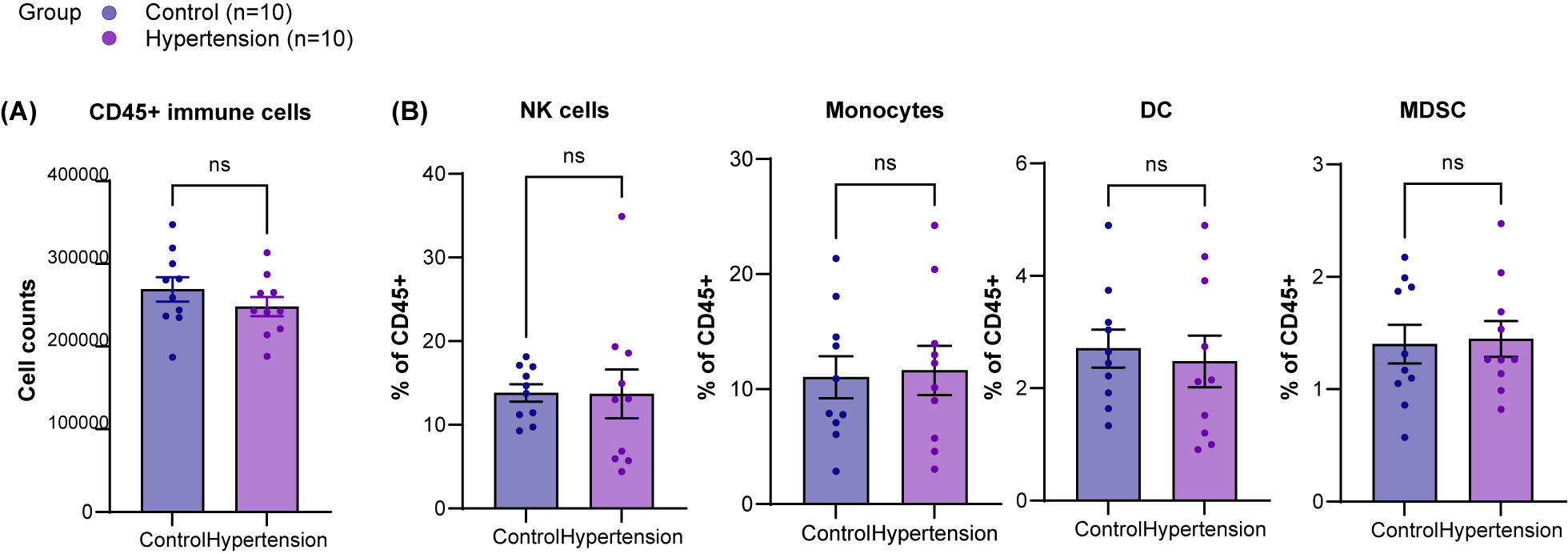

Supplement: Supplementary file 1 [file DataSheet1.zip › revised Supplementary Figures and legend/Supplementary Figure 2.tif]

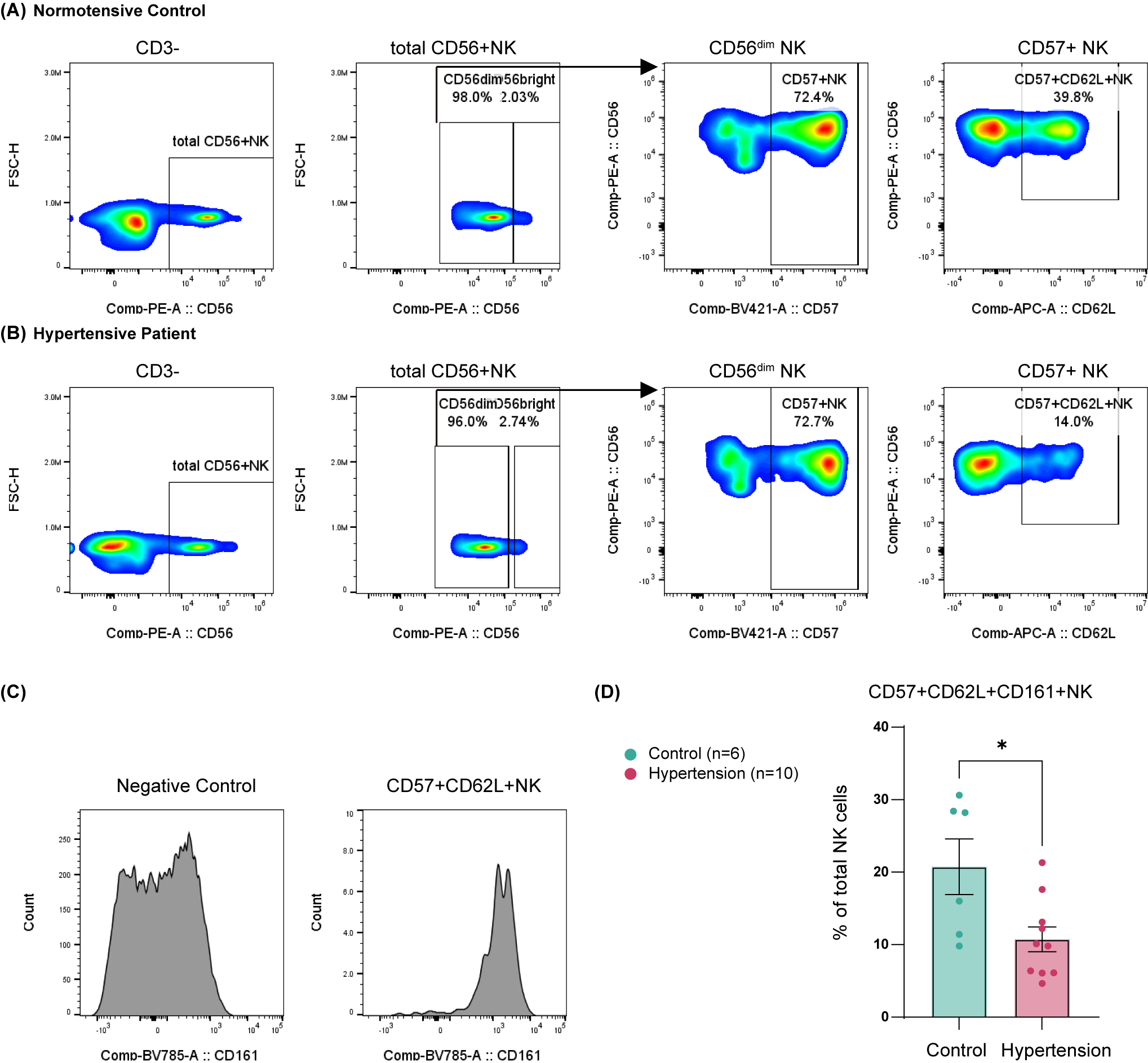

Supplement: Supplementary file 1 [file DataSheet1.zip › revised Supplementary Figures and legend/Supplementary Figure 3.tif]

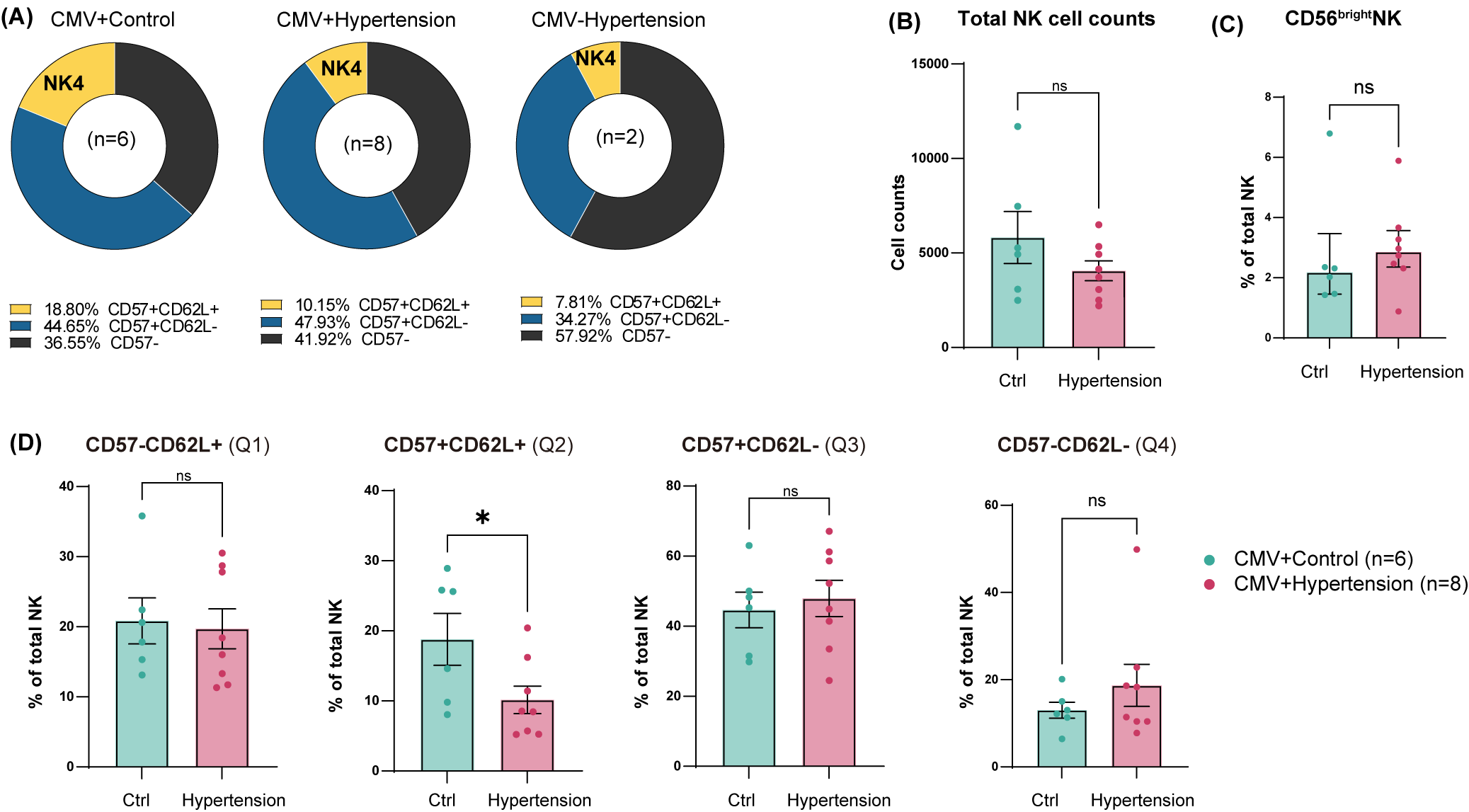

Supplement: Supplementary file 1 [file DataSheet1.zip › revised Supplementary Figures and legend/Supplementary Figure 4.tif]

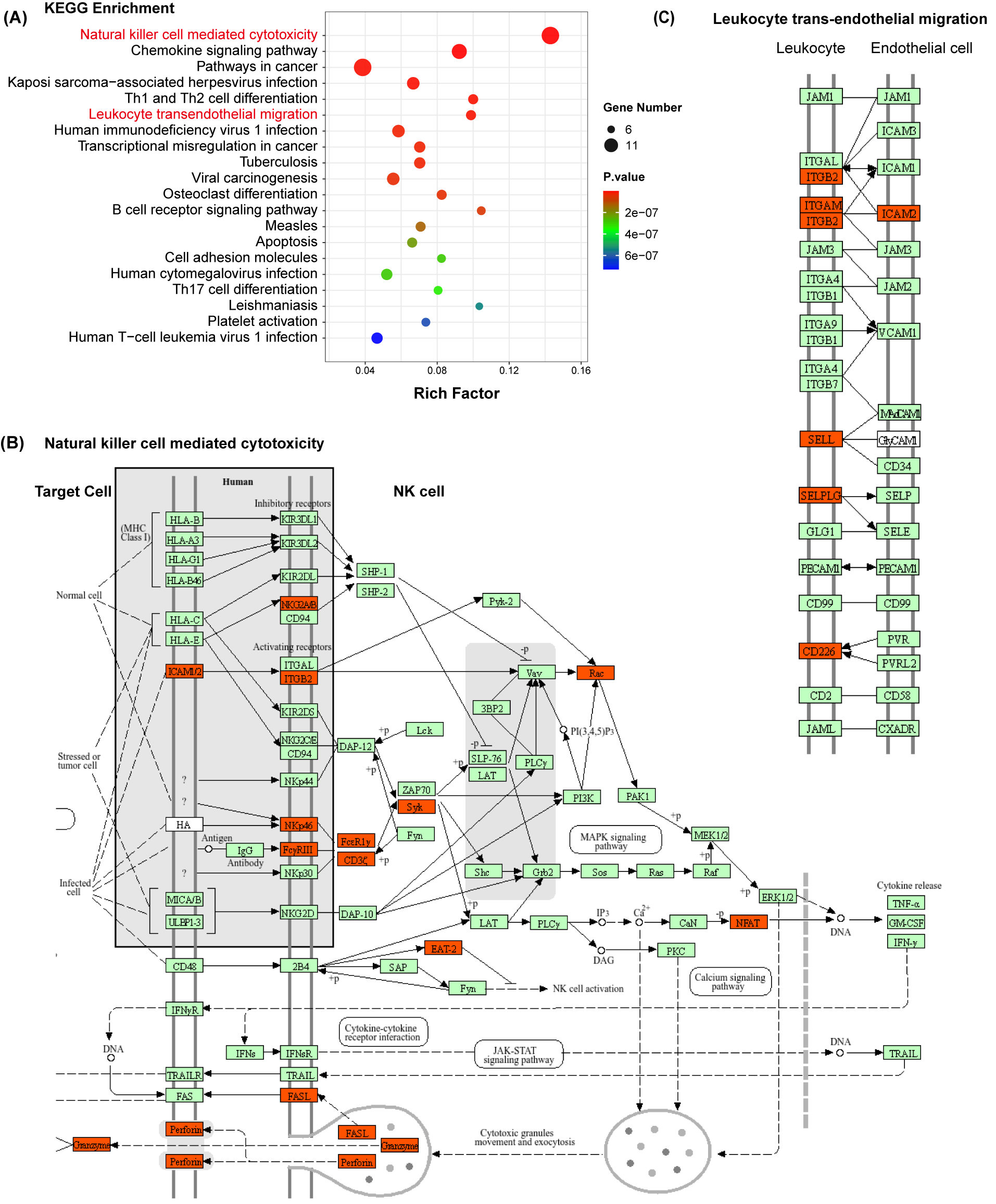

Supplement: Supplementary file 1 [file DataSheet1.zip › revised Supplementary Figures and legend/Supplementary Figure 5.tif]
